# The Bacterial Nucleoid: From Electron Microscopy to Polymer Physics—A Personal Recollection

**DOI:** 10.3390/life13040895

**Published:** 2023-03-28

**Authors:** Conrad L. Woldringh

**Affiliations:** Bacterial Cell Biology, Swammerdam Institute for Life Sciences (SILS), University of Amsterdam, 1098 XH Amsterdam, The Netherlands; c.woldringh@icloud.com

**Keywords:** electron microscopy, phase-contrast microscopy, bacterial nucleoid, DNA polymer physics, protein depletion, chromosome arms, replication bubble, active and passive DNA segregation

## Abstract

In the 1960s, electron microscopy did not provide a clear answer regarding the compact or dispersed organization of the bacterial nucleoid. This was due to the necessary preparation steps of fixation and dehydration (for embedding) and freezing (for freeze-fracturing). Nevertheless, it was possible to measure the lengths of nucleoids in thin sections of slow-growing *Escherichia coli* cells, showing their gradual increase along with cell elongation. Later, through application of the so-called agar filtration method for electron microscopy, we were able to perform accurate measurements of cell size and shape. The introduction of confocal and fluorescence light microscopy enabled measurements of size and position of the bacterial nucleoid in living cells, inducing the concepts of “nucleoid occlusion” for localizing cell division and of “transertion” for the final step of nucleoid segregation. The question of why the DNA does not spread throughout the cytoplasm was approached by applying polymer-physical concepts of interactions between DNA and proteins. This gave a mechanistic insight in the depletion of proteins from the nucleoid, in accordance with its low refractive index observed by phase-contrast microscopy. Although in most bacterial species, the widely conserved proteins of the ParABS-system play a role in directing the segregation of newly replicated DNA strands, the basis for the separation and opposing movement of the chromosome arms was proposed to lie in preventing intermingling of nascent daughter strands already in the early replication bubble. *E. coli*, lacking the ParABS system, may be suitable for investigating this basic mechanism of DNA strand separation and segregation.

## 1. Electron and Light Microscopy

**Figure 1:**
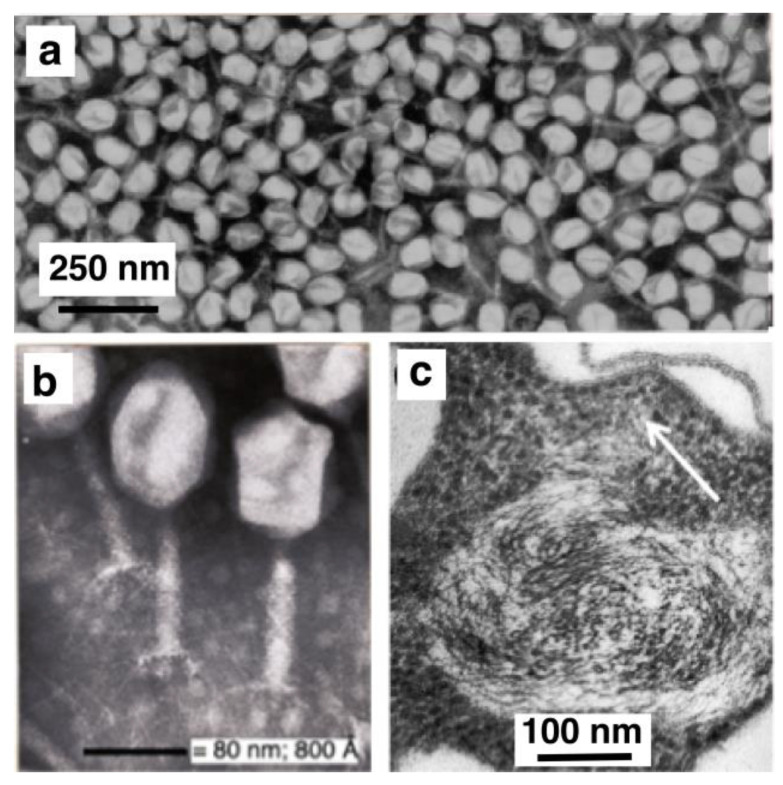
(**a**,**b**) Electron micrographs of T2r-bacteriophages, taken by N. Nanninga, 28 March 1966. The preparations were negatively stained with phosphotungstic acid and photographed at an instrumental magnification of 40,000×. (**c**) Electron micrograph of a thin section of *E. coli* K-12, fixed with osmium tetroxide according to the Ryter–Kellenberger conditions [1]. White arrow points to a presumed DNA-membrane connection.

In 1966, the preparation of T2-phages that I purified during my master’s degree program was photographed at the Laboratory of Electron Microscopy in Amsterdam by Nanne Nanninga (Figure 1a,b). The preparation was pure and satisfied the director, Dr. Woutera van Iterson, who consequently accepted me later as a Ph.D. student. I also remember how van Iterson and I looked together at the pictures of highly magnified photographs of thin sections of *Escherichia coli* cells, fixed by the Ryter–Kellenberger method [1], in which aggregated DNA threads and poly-ribosomes can be distinguished. As in the case of mesosomes in *Bacillus subtilis* (see below), van Iterson saw in the continuation of DNA threads through the cytoplasm towards the plasma membrane (arrow in Figure 1c), a confirmation of the first model for bacterial DNA segregation, in which Jacob, Brenner and Cuzin [2] proposed a connection between DNA and the plasma membrane.

Nanninga, however, was skeptical. At that time, around 1970, he was involved in applying the freeze-fracture technique to *B. subtilis* cells which were expected to contain mesosomes. These membranous organelles had no clear function, but in the thin sections studied by van Iterson, they were often seen in contact with nucleoids [3]. The shadowed replicas of unfixed, freeze-fractured *B. subtilis* cells, however, did not show any sign of mesosomes; these only appeared in the freeze-fractures when cells were previously fixed (causing permeabilization of the plasma membrane); for instance, with osmium tetroxide used for thin sectioning. A similar phenomenon seemed to occur with the variable visibility in freeze fractures of the *E. coli* nucleoid: the latter could not always be distinguished, probably due to ice crystal formation. These problems and, in addition, the difference in nucleoid appearance between osmium tetroxide and glutaraldehyde fixed [4] led Nanninga to stimulate the development of a confocal scanning light microscope [5,6], which promised to bridge the gap in resolution between electron and light microscopy.

## 2. Cell Size, Shape and Growth Models

While in the lab, the interpretation of electron micrographs of fixed or frozen cells led to emotional and unsolvable discussions about mesosomes [7], I had found, in the small library of the institute, the book: “Control of macromolecular synthesis”, by Maaløe and Kjeldgaard [8]. Especially intriguing, there was a scheme of the nucleoid and cytoplasm (see their Figure 7-1, at the end of the book [8]). During my Ph.D. program, I also tried to understand the Helmstetter–Cooper model published in 1968 [9]. There was nobody in my surroundings who knew about this model, but there was interest in my study of thin-sectioned nucleoids showing that replication and segregation went hand in hand during the cell cycle [10]. After obtaining my Ph.D. in 1974, I had the opportunity to visit the laboratory of Charles Helmstetter in Buffalo (New York), where I also met Olga Pierucci. Travelling for the first time in the US, and also meeting scientists such as Herb Kubitschek, Arthur Koch and Elio Schaechter, was an impressive and stimulating experience.

Another important stimulating event occurred when I participated at the Lunteren Lectures on Molecular Genetics of 1974. There, I showed measurements of the size and shape of *E. coli* mutant cells [11], prepared by the agar filtration method developed by Kellenberger [12,13]. After my presentation, Arieh Zaritsky approached me with a clear message: “We have to meet and talk about cell shape!”. Having already received his Ph.D. at the laboratory of Bob Pritchard (Leicester, UK), Arieh seemed to understand the recent physiological experiments of Maaløe and Kjeldgaard, as well as the Helmstetter–Cooper model that described the coordination between chromosome replication and cell division [14,15]. This was the beginning of a still-lasting cooperation [16] that started with learning how to culture *E. coli* cells under steady state conditions while analyzing shape changes during a nutritional shift-up of cells prepared by agar filtration and understanding the distinction between the two completely different physiological states of “thymine starvation” and “thymine limitation” [17].

Together with Nanne Nanninga, Arieh Zaritsky, Bob Rosenberger, Norman Grover, Wim Voorn and Luud Koppes, the electron microscope measurements of fixed and air-dried cells were compared to growth models that predicted the observed shape of length distributions; we discussed cell elongation modes (linear with a rate doubling or exponential), shape changes and correlations between cell cycle events such as initiation of DNA replication (derived from radio-autograms) and initiation of cell constriction, the so-called *C+D-T* period. In 1993, Voorn, Koppes and Grover, remarked in a short paper [18] that a newly developed “incremental-size model” could not be rejected. Previously, the occurrence of “a constant size increment” during the *C+D-T* period was mentioned in Figure 6 of Koppes et al. [19] and Figure 1 of Koppes and Nanninga [20], suggesting a strong positive correlation between the events of initiation of DNA replication and initiation of cell constriction.

More than 20 years later, the same model was going to basically cause an explosion of studies [21,22]. This revival of the model can be ascribed to Suckjoon Jun, who gave it the name “adder”, writing: “The beauty of this “adder” is that it automatically ensures size homeostasis” (see also the Movie S1, “Size convergence by adder principle, related to Figure 3” in [23]). According to this now widely accepted adder model, based on measurements of living cells, often grown in microfluidic devices, cells do not sense their size (sizer model) nor their age (timer model), but add a constant size, between birth and division, that is independent of birth size. Whether and how cells could “sense” a constant size increment in large and small newborn cells is still unknown. However, measuring the amount of DNA in large and small prospective daughter cells in fast-growing *E. coli* cells [24] showed an increased amount of DNA (20% higher) in large siblings. This observation is in agreement with the prediction that large newborns initiate DNA replication earlier [25]. In addition, nucleoid segregation was found to be advanced in these larger prospective daughter cells, allowing them to divide earlier, as to be expected from the adder model. Confirmation of this adder-like behavior based on DNA replication and segregation has to await visualization of differently sized siblings in quantitative time-lapse experiments, as performed by the group of Jaan Männik [26].

## 3. Nucleoid Occlusion and Transertion

During his short-term EMBO-fellowship visit to Amsterdam in 1977, Arieh Zaritsky proposed to organize together with Nanne Nanninga the first EMBO workshop on bacterial duplication. It was held in 1980 in Noordwijkerhout (The Netherlands) with leaders in the field of bacterial physiology, such as Donachie, Grover, Helmstetter, Koch, Kubitschek, Maaløe, Messer, Pierucci, Pritchard and Schwarz.

Arieh organized the second workshop in Sede Boqer (Israel) in 1984, which I attended after enjoying a sabbatical leave in the lab of Jim Walker at the University of Texas at Austin. While continuing our cooperation, the study of populations of cell division mutants in Amsterdam was greatly facilitated by Norbert Vischer, who listened to our wishes for measuring cell properties and who translated them into practical software for image analysis and visualization of results [27]. This also enabled us to develop an interactive cell cycle simulation (CCS) program [28], which was used for decades to predict behavior of emerging cell-cycle mutants and to teach students the Helmstetter–Cooper model [29].

**Figure 2:**
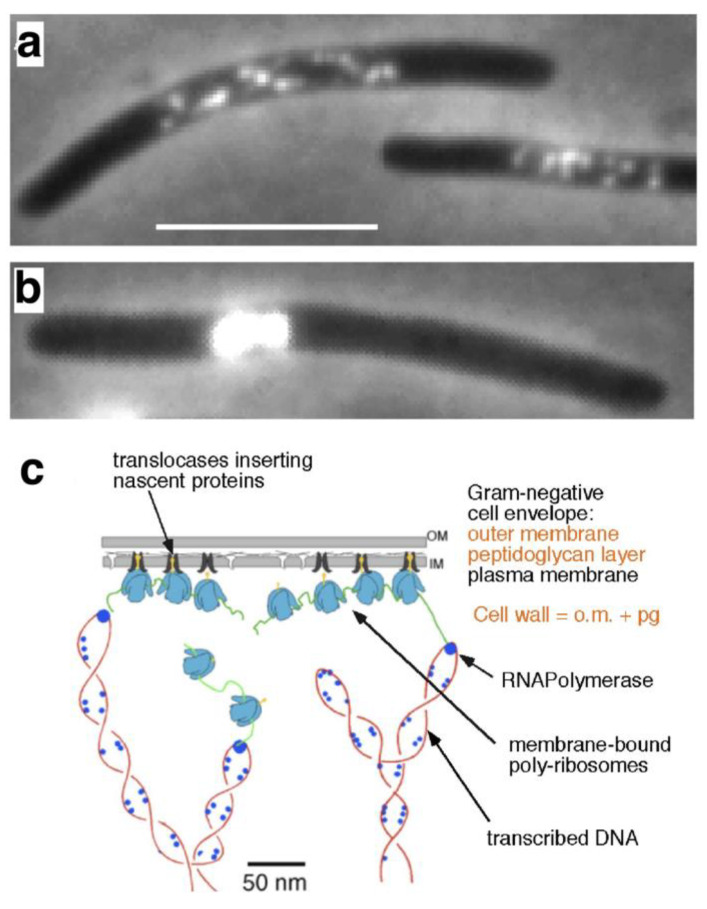
(**a**,**b**) Filaments of *E. coli dnaX* (Ts) grown at restrictive temperature (42 °C) for several mass doublings, fixed with 0.1% osmium tetroxide and stained with DAPI. (**a**) While DNA synthesis stops immediately, cells continue to grow, forming SOS-filaments. During elongation, the original nucleoid is pulled apart into small lobules. (**b**) Upon growth inhibition with 300 µg/mL chloramphenicol the DNA lobules re-compact into a confined region [34]. Bar in (**a**) also holds for (**b**) and represents 5 µm. (**c**) Schematic representation of the “transertion model” [37].

It was also during this period that, together with Nanninga, Wientjes and Zaritsky and Ph.D. students (Egbert Mulder, Marko Roos, Peter Taschner, Frank Trueba, Jacques Valkenburg and Joop van Helvoort), the concept of “nucleoid occlusion” was developed [30]. The term was coined by Larry Rothfiel and originally applied to the idea that transcriptional activity around the nucleoid occludes the increased rate of peptidoglycan synthesis necessary to initiate constriction [31]. Along with the ideas of Vic Norris [32], our observations on *E. coli* nucleoids and quantitative measurements by Evelien Pas, Peter Huls and Norbert Vischer resulted in the formulation of the “transertion model”. Observations of an expansion of non-replicating nucleoids by active protein synthesis [33], their compaction and fusion by inhibition of protein synthesis with chloramphenicol [34] and re-segregation after release from inhibition that occurred faster than cell elongation [35] led to the proposal that coupled transcription–translation–translocation of envelope proteins (transertion) could play an active role in DNA segregation (Figure 2).

However, about 20 years later, this idea could be falsified with the help of constructs made by Flemming Hansen (Denmark). Because the positioning of the left (L) and right (R) chromosome arms during replication showed a similar ordering pattern in either growing cells (e.g., L-ori-R L-ori-R or L-ori-R-R-ori-L), or during run-off DNA replication in protein-synthesis inhibited cells, transertion could not play a role in the mere movement of the chromosome arms [36]. This movement was proposed to be the passive result of DNA synthesis itself rather than of active protein synthesis (see Section 5). It should be noted, however, that active transertion influences the ordering pattern of the left and right chromosome arms and is still required for separation and movement of the entire daughter nucleoids into the prospective daughter cells [36].

## 4. Physical DNA Model

**Figure 3:**
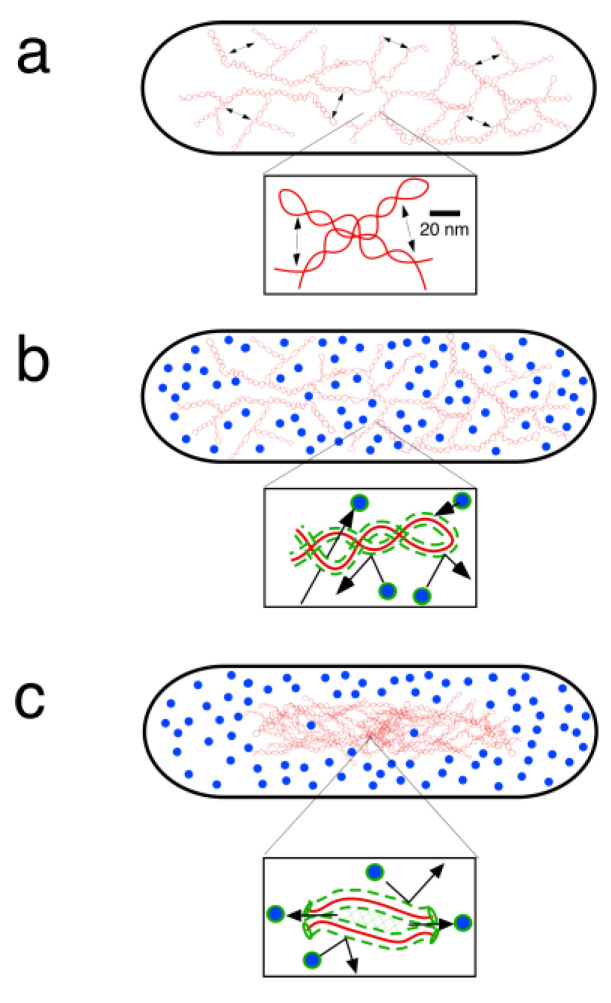
Description of DNA–DNA and DNA–protein interactions that contribute to a phase separation between nucleoid and cytoplasm. (**a**) When the supercoiled DNA is introduced into an empty cell, free energy is stored because the expansion of the network due to the colliding supercoil segments (indicated by double arrows) has to be overcome. (**b**) When, in addition, many proteins are introduced into the cell, a free energy increase occurs that is associated with the cross-interactions between proteins and the DNA helix, both exhibiting an electrostatic depletion radius, indicated by the green zone around the DNA chains. This cross-interaction energy overwhelms the self-interaction energy in (**a**), leading to an unstable situation. (**c**) To minimize the free energy of the total system, a phase separation is established in which the DNA is compacted in a smaller volume with decreased protein-DNA cross-interactions. The latter is obtained because overlapping depletion zones (green areas) between the DNA strands in the compacted nucleoid, squeeze-out (deplete) proteins, resulting in a lower protein concentration and in ~30% reduction in nucleoid density as shown in phase-contrast images [38,42].

However, what about the remaining controversy concerning the dispersed or compact organization of DNA in the bacterial nucleoid? During and after my Ph.D., I remained fascinated by Figure 7-1 of Maaløe and Kjeldgaard [8] and I was glad that, with the help of Theo Odijk, we could make a similar figure, based on our measurements of nucleoid volume [38], on recent data of macromolecular concentrations in *E. coli* cells [39] and on Odijk’s free-energy approach of calculating the excluded-volume interactions between soluble proteins and DNA [40]. This so-called depletion theory (see explanation in [41]) formulates the free energy of the system that tends to reach equilibrium by minimizing its total free energy. The theory considers the free energy of self-interactions between DNA supercoils and of cross-interactions between DNA and soluble, cytoplasmic proteins and predicts a phase separation between nucleoid and cytoplasm as described in Figure 3.

Together with colleagues such as the late Michiel Meijer [43], Paul Sloof [44] and, subsequently, with Suckjoon Jun [45], we finally succeeded in reproducibly liberating nucleoids from *E. coli* spheroplasts by osmotic shock and in measuring the size of free-floating nucleoids under different crowding conditions (e.g., PEG; see Figure 9C in [46]). We also calculated the very small diffusion coefficient of a DNA region near *oriC* in isolated nucleoids [47] and, with Steve Elmore, Michiel Müller, Norbert Vischer and Theo Odijk, also in living cells [48].

Finally, a model for the bacterial nucleoid could be developed [49] (see [45] for microfluidics experiments). In the model, the DNA is represented by branched supercoils, partly relaxed through association with DNA-binding proteins and cross-linked by a substantial number of physical entanglements and/or proteins into a homogeneous, core-less network, without any sign of a “highly ordered structure”, as often proposed. While during osmotic shock of the spheroplasts, the nucleoids enlarge about 100 fold in volume, the liberated and DAPI-stained nucleoids expand further under continued UV irradiation (Figures 2 and 4 in ref. [49]). 

In all our experiments, a distinctive substructure of granules (diameter ~ 2 µm) became visible during this expansion, showing Brownian motion. It is tempting to speculate that these granules correspond with the uncrosslinked blobs calculated by Odijk to have a radius of gyration of ~0.9 µm (see Appendix B in [49]).

## 5. Segregation of Chromosome Arms

**Figure 4:**
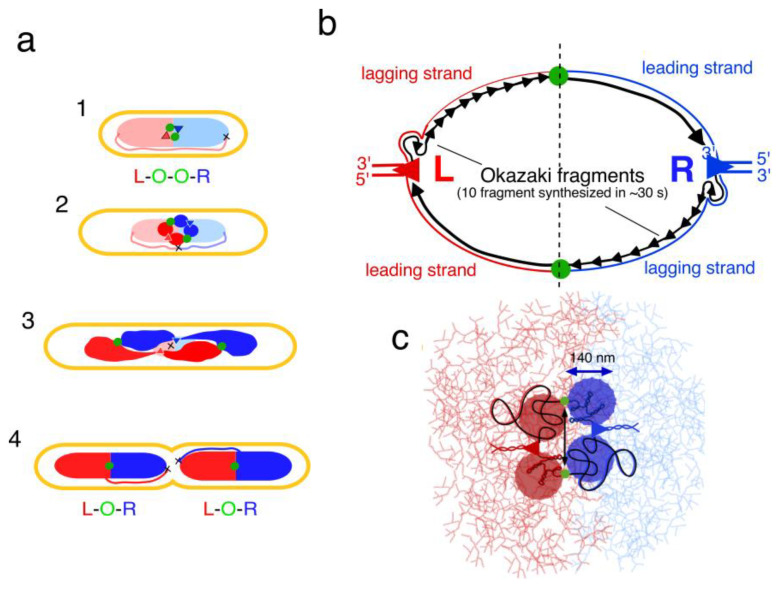
Segregation of chromosome arms in *E. coli*. (**a**) In newborn cells, the two replichores (red and blue) occupy separate regions within the nucleoid as documented in [50,51]. Colored triangles are the replisomes duplicating the left (L; red) and right (R; blue) chromosome arms. The duplicated origins (green circles) lie in between, giving the pattern L-O-O-R (panel 1). Note the replacement of the unreplicated, parental nucleoids (light colors) by the newly synthesized DNA (dark colors). The two pairs of replichores that are synthesized in the replication bubble are assumed not to become mixed or entangled, but to form four individual domains (dark-colored circles in panel 2; see text) in a transversal arrangement [53,54]. De novo DNA synthesis expands and rearranges the domains (panel 3); they end up as four separate domains in each half of the two daughter nucleoids (panel 4). (**b**) Schematic representation of the replication bubble or orisome [55], ~30 s after initiation, when 10 Okazaki fragments have been synthesized. Physical differences between leading and lagging strands are proposed to prevent the mixing of the nascent strands (see text). (**c**) Hypothetical representation of the same replication bubble as in (**b**), consisting of four enlarging blobs (filled circles) that do not mix. The length of DNA (~10 µm) synthesized in 30 s (see (**b**)) is drawn as a spherical blob with a volume of 0.0014 µm^3^ (cf. volume non-replicating nucleoid of 0.24 µm^3^; calculation to be published elsewhere). Due to tethering of the replisomes to parental DNA, the origins between the nascent domains are pushed apart (indicated by double arrow). During continued DNA synthesis the blobs fold into enlarging domains (see (**a**), panel 2 and 3).

However, how do daughter strands, newly synthesized in such a seemingly homogeneous network (see Figure 5 in [49]), separate and remain unmixed? Although *E. coli* lacks the ParABS system, measurements of fluorescently tagged gene loci showed [50,51] that the two newly synthesized chromosome arms end up as individual domains in different halves of the two daughter nucleoids (Figure 4a, panel 4). This arrangement could be explained by assuming that already at initiation of DNA replication, each of the two replisomes in the replication bubble synthesizes daughter strands that do not mix because of their physical differences (Figure 4b,c). These differences could arise because the leading strands become supercoiled, while in the lagging strands the Okazaki fragments have to first be ligated (Figure 4b). It is proposed that the four nascent strands exclude each other and fold into four individual blobs, screened-off from each other. Their intermingling would require extra excluded-volume interactions and thus, extra free energy (loss of entropy); as a result, the four nascent strands will remain separated in a minimum energy situation. During continued *de novo* DNA synthesis, the blobs may fold into four enlarging and separate domains stabilized by newly recruited nucleoid-associated proteins (NAPs; see review [52]) required for gene expression (Figure 4c).

By comparing the time of initiation and the time of duplication of fluorescent *oriC*-GFP spots in *E. coli,* it became evident that newly synthesized origins separate soon after their duplication [48], without a significant period of “cohesion”. It should be noted, however, that in several laboratories, data were obtained that were interpreted to indicate a period of cohesion [56,57,58]. An early separation, not necessarily incompatible with a transient cohesion period, is to be expected if the replicated origin-DNA in the replication bubble is more mobile than the two replisomes. This could be the case if the replisomes remain tethered to the compact mass of unreplicated parental DNA which they are reeling in. Tethering of the replisomes will force the duplicated origins to move apart (double arrow in Figure 4c). The expanding domains, enlarging through de novo DNA synthesis, will rearrange themselves in the long axis of the rod-shaped cell towards the two halves of the daughter nucleoids in a segregation process that requires no other driving force than continued replication (Figure 4a, panel 3). Similar ideas were expressed by Suckjoon Jun [45,59,60]. The hypothesis that segregation is merely driven by the process of de novo DNA synthesis and accumulation was previously proposed by Alan Grossman [61].

## 6. Conclusions

Studies of bacterial DNA organization and segregation exhibit two different views: either resolution and movement of replicated daughter strands is performed by a dedicated, active process based on DNA loop extrusions through structural maintenance of chromosome (SMC) complexes [62], or by the passive process of de novo DNA synthesis, as described here. If, in the replication bubble (Figure 4b), initial intermingling of the newly synthesized DNA strands would occur, it is to be expected that the entanglements could only be resolved with an elaborate mechanism of topoisomerases and SMC proteins [62]. However, the different physical properties of the nascent leading and lagging daughter strands (Figure 4b), together with different gene expression activities between the two replichores, could prevent the mixing of the four daughter strands right from the beginning. In that case, the secret of segregation lies in the build-up of the replication bubble: if no initial mixing occurs due to their different physical properties, they will become confined in four individual blobs (Figure 4c) that expand into individual domains (Figure 4a). A similar build-up of replication bubbles and early separation of strands could occur in eukaryotic chromosomes [63].

When a more detailed quantification of the number of proteins involved in the replication bubble will become available, calculations of the free energy state of the proposed four domains, as performed by Odijk for the whole nucleoid (compare Figure 3), could become possible. Such calculations might support the above proposal of passive DNA strand exclusion and formation of the four domains (Figure 4c) that gradually replace the parental nucleoid.

So far, microscopic observations have not given any indication for the existence of these domains. Further developments in spatial light interference microscopy [64], or digital holographic microscopy combined with optical diffraction tomography [65] and improved labeling techniques for nascent DNA strands [66] will be necessary to evaluate the above hypothesis of the four blobs initially created in the early replication bubble and developing into the four domains that end up in different halves of the two daughter nucleoids (Figure 4a).

## Data Availability

Not applicable.
